# Population Diversity of Rice Stripe Virus-Derived siRNAs in Three Different Hosts and RNAi-Based Antiviral Immunity in *Laodelphgax striatellus*

**DOI:** 10.1371/journal.pone.0046238

**Published:** 2012-09-28

**Authors:** Yi Xu, Lingzhe Huang, Shuai Fu, Jianxiang Wu, Xueping Zhou

**Affiliations:** 1 State Key Laboratory of Rice Biology, Institute of Biotechnology, Zhejiang University, Hangzhou, China; 2 State Key Laboratory for Biology of Plant Diseases and Insect Pests, Institute of Plant Protection, Chinese Academy of Agricultural Sciences, Beijing, China; University of Wisconsin-Milwaukee, United States of America

## Abstract

**Background:**

Small RNA-mediated gene silencing plays evolutionarily conserved roles in gene regulation and defense against invasive nucleic acids. Virus-derived small interfering RNAs (vsiRNAs) are one of the key elements involved in RNA silencing-based antiviral activities in plant and insect. vsiRNAs produced after viruses infecting hosts from a single kingdom (i.e., plant or animal) are well described. In contrast, vsiRNAs derived from viruses capable of infecting both plants and their insect vectors have not been characterized.

**Methodology/Principal Findings:**

We examined Rice stripe virus (RSV)-derived small interfering RNAs in three different hosts, *Oryza sativa*, *Nicotiana benthamiana* and a natural RSV transmitting vector *Laodelphgax striatellus*, through deep sequencing. Our results show that large amounts of vsiRNAs generated in these hosts after RSV infection. The vsiRNAs from *N. benthamiana* and *L. striatellus* mapped equally to the genomic- and antigenomic-strand of RSV RNAs. They showed, however, a significant bias in those from *O. sativa*. Furthermore, our results demonstrate that the number and size distributions of vsiRNAs in the three hosts were very different. In *O. sativa* and *N. benthamiana*, most vsiRNAs were mapped to the discrete regions in the RSV genome sequence, and most of the vsiRNAs from these two hosts were generated from RSV genomic RNAs 3 and 4. In contrast, the vsiRNAs identified in *L. striatellus* distributed uniformly along the whole genome of RSV. We have also shown that silencing Agronaute 2 in *L. striatellus* enhanced RSV accumulation in this host.

**Conclusions/Significance:**

Our study demonstrates that the core RNA-induced gene silencing (RNAi) machinery is present in *L. striatellus*. We also provide evidence that the RNAi-mediated immunity against RSV is present in *L. striatellus*. We propose that a common small RNA-mediated virus defense mechanism exists in both helipterum insects and plants, but the vsiRNAs are generated differentially in different hosts.

## Introduction

Approximately 80% of the plant viruses are known to be transmitted through insect vectors [1]. This insect dependent transmission can be divided into non-persistent, semi-persistent and persistent transmission, depending primarily on the length of successful transmission of virus to host plant [2]. Studies using *Drosophila* demonstrated that virus infection in insect could cause production of virus-derived small RNAs (sRNA) and these sRNA molecules could then induce specific antiviral immunity in insect through a mechanism known as RNA interference (RNAi) [3], [4], [5].

Numerous studies have indicated that RNAi-based antiviral response is one of the key antiviral strategies identified in plant and invertebrate [6]. The key element involved in the RNAi-based antiviral response is the virus-derived small interfering RNAs (vsiRNAs) processed by RNaseIII-like enzymes, also known as Dicers, from double-stranded RNAs (dsRNAs) or structured single-stranded RNAs (ssRNAs). The vsiRNAs are then recruited into the RNA-induced silencing complex (RISC) and target viral RNA molecules for degradation in a sequence-specific manner. Among the reported plant species, *Arabidopsis thaliana* and *Oryza sativa* are the most well studied species for RNAi [7], [8]. The Dicer-like (DCL) protein 1 (DCL1) and Agronaute 1 (AGO1) are known to be responsible mainly for production of 21-nt microRNAs (miRNA), and DCL4 targets primarily positive-stranded viral RNAs and produces 21-nt vsiRNAs. DCL2 was reported to rescue silencing against RNA viruses when DCL4 was inactivated or suppressed, and was responsible for producing 22-nt vsiRNAs [9], [10], [11], [12].

In *Drosophila melanogaster*, AGO1 and AGO2 are known to recruit miRNAs and siRNAs, respectively. The role of Dicer-2 (Dcr2) in antiviral reaction is to identify dsRNAs accumulated in virus infected cells and processes them into 21-nt siRNA duplexes followed by 3′ end methylation by the Hen1 methyltransferase. These 21-nt siRNAs interact with AGO2 in the presence of R2D2, a dsRNA binding protein, and several other proteins to form the RISC complexes for targeting more viral RNAs [3], [4], [13]. More recently *in vitro* synthesized double-strand RNAs were utilized to determine gene function in insect through either micro-injection or feeding methods [14]. Results from numerous studies have indicated that RNAi pathway exists in insects.

In the past decade Rice stripe disease has become a major threat to rice production in the southern and central parts of China. This disease is caused by Rice stripe virus (RSV), an RNA virus found in China and many other countries in the East Asia [15]. After RSV infection, rice plants often show chlorosis and necrosis in their newly developed leaves followed by stunting of the plants [16]. RSV is a member of the genus *Tenuivirus* and has thin filamentous particles. Genome of RSV consists of four single-stranded RNA segments known as RNAs 1, 2, 3 and 4; RNA1 is negative-sense and RNAs 2, 3 and 4 are ambisense [17]. RSV is transovarially transmitted by *Laodelphgax striatellus* (small brown planthopper, SBPH) in a circulative-propagative manner [18], [19]. In addition to infect plant species in the family *Gramineae*, RSV can also replicate in *L. striatellus* and *Nicotiana benthamiana*, a widely used experimental host for many plant viruses [20].

VsiRNAs were previously identified in RSV-infected rice leaves and their role in host defense against RSV infection was proposed [21]. However, information on RSV vsiRNAs population diversity in RSV-infected *N. benthamiana* and *L. striatellus* was lacking. Using deep sequencing and dsRNA injection technology, we demonstrate the presence of RSV vsiRNAs in both *N. benthamiana* and *L. striatellus* Our results also indicate the potential existence of RNAi-mediated immunity against RSV infection in *L. striatellus*, a member of *Hemipteran* that transmits about 55% of the known plant viruses.

## Results and Discussion

### RSV-derived vsiRNAs in *L. striatellus*, *O. sativa* and *N. benthamiana*

**Figure 1:**
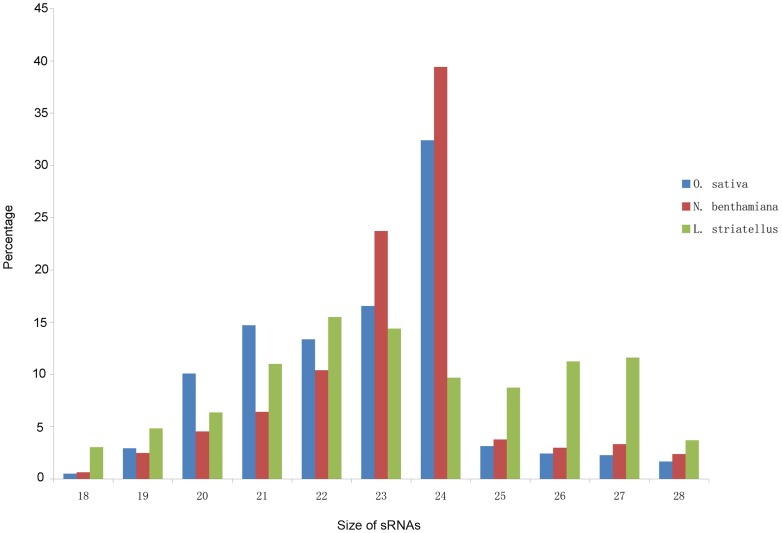
Size distribution of total sRNAs in libraries prepared from RSV infected *O. sativa*, *N. benthamiana* and *L. striatellus*. The sRNAs (18∼28-nt) generated in *O. sativa*, *N. benthamiana* and *L. striatellus* are shown in blue, red and green, respectively.

Illumina deep sequencing technology was used in this study to identify vsiRNAs in RSV-infected *L. striatellus*, *O. sativa* and *N. benthamiana*. Analysis of siRNA libraries from these three hosts showed that most siRNAs from *O. sativa* and *N. benthamiana* were 18 to 24-nt in length and among them the 24-nt class is the most dominant class, accounting for 32.4% siRNA in *O. sativa* and 39.4% in *N. benthamiana*, respectively (Figure 1). This result is similar to that found in sRNA libraries derived from other plant species [22], [23]. In the sRNA library from *L. striatellus*, the 22-nt class sRNAs represented the major sRNA class, and the other major sRNA class was 26 to 27-nt, a sRNA class similar to the Piwi-interacting RNA (piRNA). The piRNAs are small RNA molecules derived from non-coding RNA molecules and are also involved in gene silencing, particularly in silencing transposons [24], [25].

**Figure 2:**
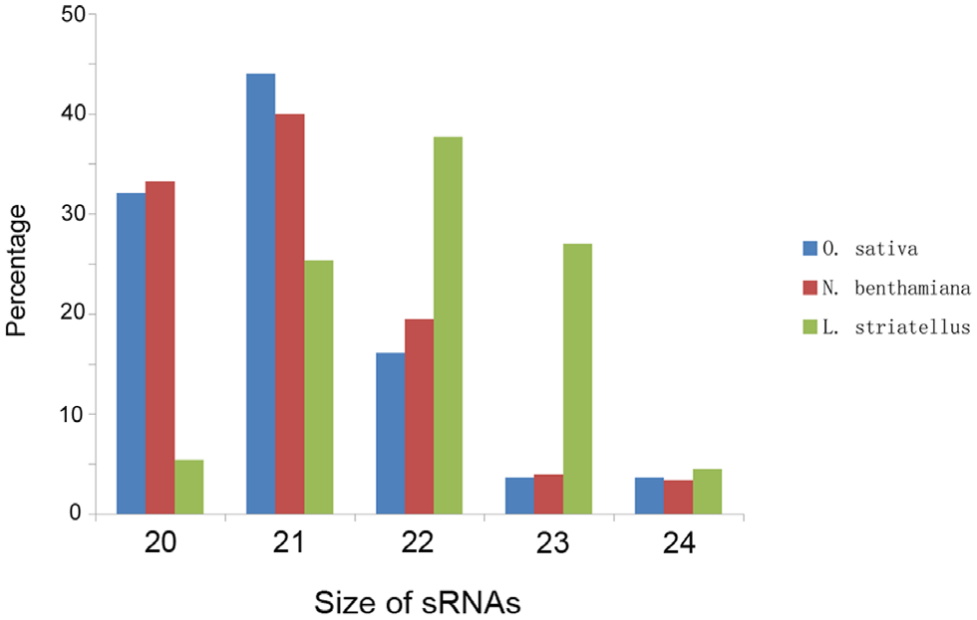
Distributions of sRNA sequences matching RSV genomes from RSV-infected *O. sativa*, *N. benthamiana* and *L. striatellus*. The percentage of vsiRNA (20∼24-nt) from *O. sativa*, *N. benthamiana* and *L. striatellus* are shown in blue, red and green, respectively.

**Figure 3:**
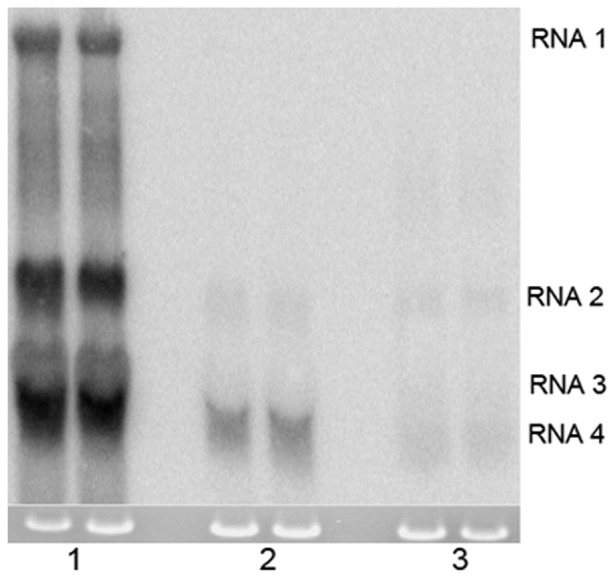
Accumulation of RSA genomic RNAs in infected *O. sativa* (1), *N. benthamiana* (2) and *L. striatellus* (3). Fifteen µg total RNA extracted from RSV-infected *O. sativa*, *N. benthamiana* and *L. striatellus* were used for the Northern blot assay. The ethidium bromide-stained 18s rRNA was shown as the RNA loading control.

To determine whether some sRNAs identified in these three hosts were from RSV genomes, we aligned sRNA sequences with the RSV genomic and antigenomic RNA sequences. Results show that there is large number of RSV-derived sRNAs (vsiRNAs) in these libraries, and of these vsiRNAs, *O. sativa* is the main source followed by *N. benthamiana* and then *L. striatellus* (Table 1 and Figure 2). Northern blotting show that the virus level in rice is higher than that in *N. benthamiana*, and the virus level in SBPH was the lowest (Figure 3). This finding suggests that the accumulation of vsiRNAs may correlate with the accumulation levels of RSV RNAs in these hosts. Alternatively the secondary siRNAs may play a role in increasing siRNAs accumulation, mediated by RNA-dependent RNA polymerases (RDRs). Although RDRs were identified in *C. elegans* [26], [27], [28], it has not been reported in insects or vertebrates. The absence of RDRs in *L. striatellus* was also confirmed by searching three transcriptome data from *L. striatellus* (data not show). Whether RDR sequence can be found in the whole genome of planthoppers requires further investigation.

**Table 1:** Summary of Illumia deep sequencing data.

	Rice	*N. benthamiana*	SBPH
Reads after removing adaptor	1291515	2309997	1489712
Reads matching viral genome	198936	52119	15537
Reads matching Genomic strands	129654	28197	8719
Reads matching Antigenomic strands	69282	23922	6818
Total 21-nt vsiRNA	87574	20834	3702
Total 22-nt vsiRNA	32063	10143	5502
Total 23-nt vsiRNA	8264	2050	3946
Total 24-nt vsiRNA	7242	1772	658
RNA2 locus 778–801	388	497	0

The numbers of 20 to 24-nt vsiRNAs identified in these three hosts are shown in Table 1. The 20∼24-nt vsiRNAs were largely seen in *O. sativa* and *N. benthamiana*, while the 21 to 23-nt vsiRNAs were mainly seen in *L. striatellus*. This observation suggests that the pathway for 24-nt vsiRNAs generation may not present in *L. striatellus*, and thus different mechanisms of vsiRNA production may exist in plant and insect. In *O. sativa* and *N. benthamiana* the 21-nt vsiRNA is the main class of vsiRNA and in *L. striatellus* the 22-nt vsiRNA is the most dominant class. The 22-nt vsiRNAs identified in *L. striatellus* may be cleaved by unidentified dicers and these are capable of targeting viral RNAs for silencing. Indeed, an enhanced susceptibility to Beet curly top virus (BCTV) and CaLCuV infection was observed in the dcl2-deficient *Arabidopsis*, and 22-nt siRNAs were identified in the BCTV- and CaLCuV-infected dcl2-deficient *Arabidopsis* [29], [30]. Further investigation is needed to elucidate the role of 22-nt vsiRNAs in targeting RSV for silencing in *L. striatellus*.

**Figure 4:**
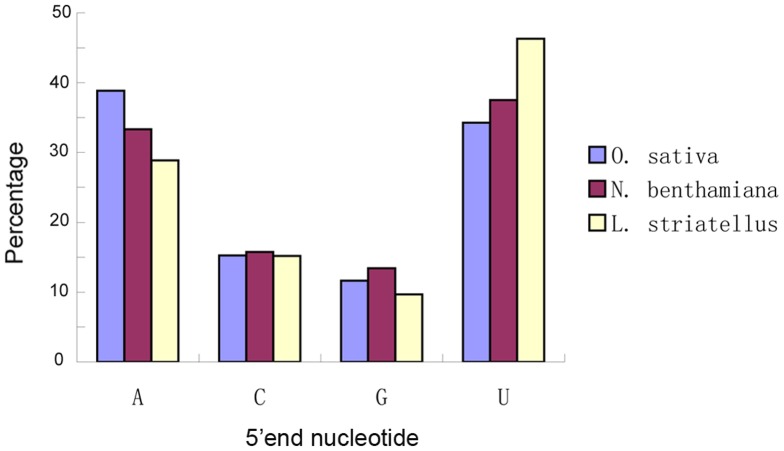
Relative frequency of 5′ terminal nucleotide of vsiRNAs. The vsiRNAs derived from RSV-infected generated in *O. sativa*, *N. benthamiana* and *L. striatellus* are shown in blue, red and green, respectively.

**Figure 5:**
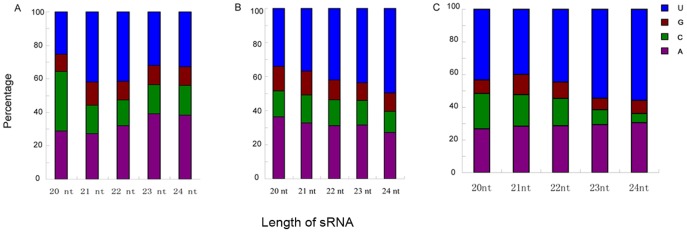
Relative frequency of 5′ terminal nucleotide for 20 to 24-nt vsiRNAs. A, B and C represent RSV-derived vsiRNAs from *O. sativa*, *N. benthamiana* and *L. striatellus*, respectively.

Previous studies have indicated that in plant, the 5′-terminal nucleotides of sRNAs controls the binding of siRNAs to specific Ago complexes [8], [31], [32]. In this study our bioinformatic analysis revealed a preferential use of uridine (U) and adenosine (A) residues, as compared to cytosine (C) and guanidine (G), by vsiRNAs in all three small RNA libraries (Figure 4). The preferential use of C was identified for the 20-nt vsiRNA derived from *O. sativa* and U for the 24-nt vsiRNA from *N. benthamiana*. This finding suggests the involvement of different Ago complexes in different plant species for vsiRNAs (Figure 5). Majority 21- and 22-nt vsiRNAs identified in plants in this study showed a strong bias of sequence beginning with a 5′-U. This is consistent with the role described for Ago1 in defending against RNA viruses in plant [33], [34]. In fruit fly double-stranded small RNAs with perfect sequence match are favorable to enter the Ago2 complex, whereas small RNA duplexes with bulges favor to bind with Ago1 [35]. A recent report demonstrated that small RNAs in *Drosophila* were sorted for Ago1 or Ago2 according to their duplex structures and the identity of their first nucleotide [36]. The 5′-terminal nucleotides of vsiRNAs from *O. sativa*, *N. benthamiana* and *L. striatellus* were similar, indicating that these nucleotides may have role(s) in targeting vsiRNAs to Ago complexes. Isolation of Ago complexes from *L. striatellus* and other insect vectors, and determination of small RNAs associated with these complexes will provide us more information Ago complexes and their functions on vsiRNA generation.

### Identification of vsiRNA hot spots in RSV genome

**Figure 6:**
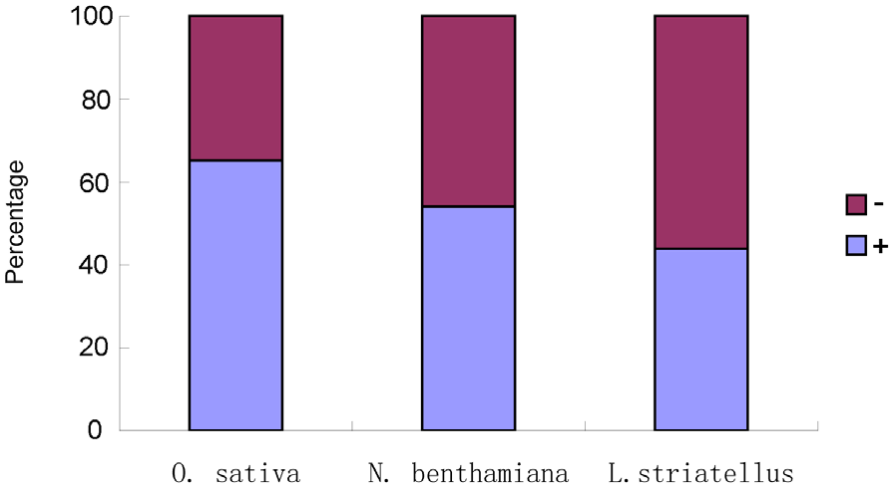
Statistical analysis of vsiRNAs mapped to the RSV genomic (−) or antigenomic (+) sequences. In each species, red color represents the (−) sRNA, and blue color represents the (+) sRNA.

**Figure 7:**
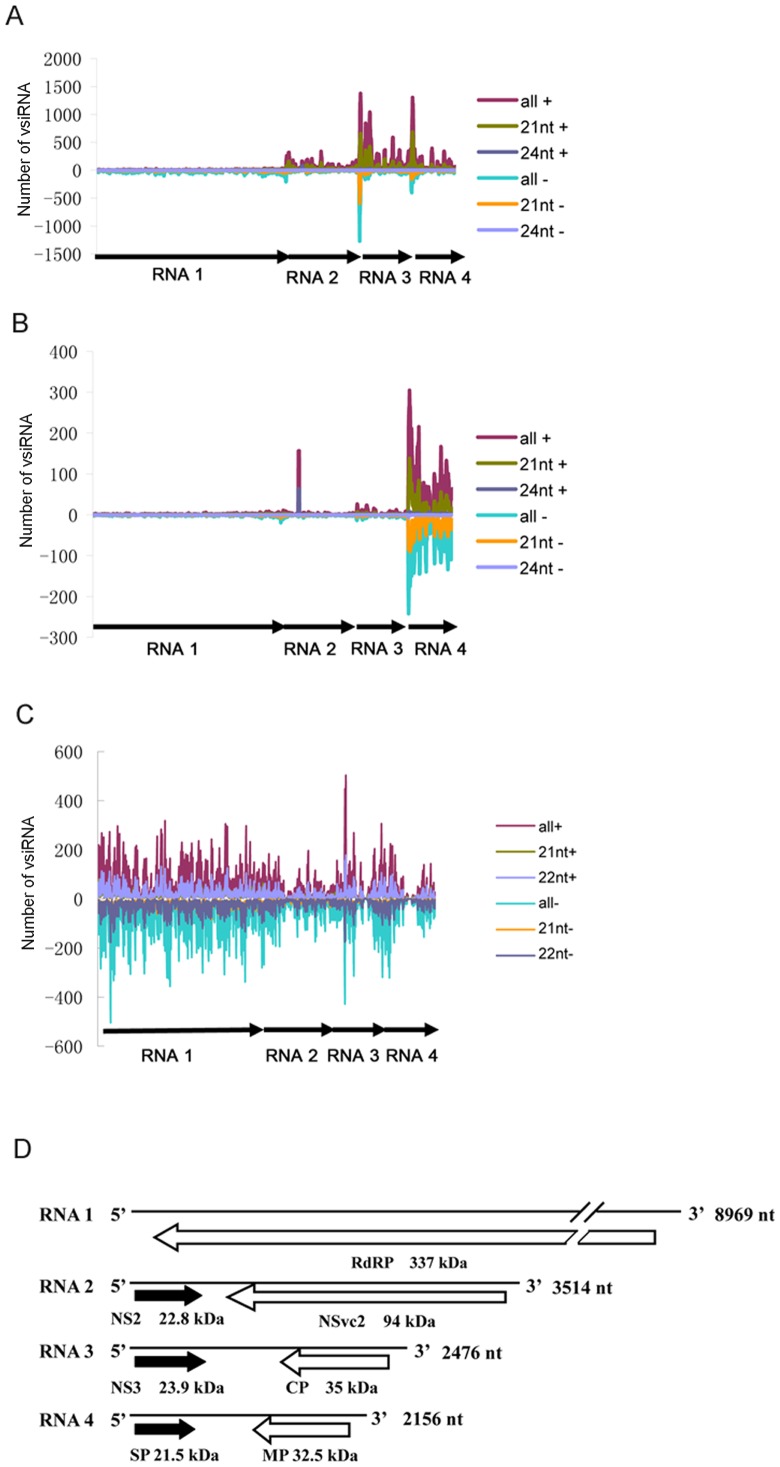
Profile of RSV-derived vsiRNAs from *O.sativa* (A), *N. benthamiana* (B) and *L. striatellus* (C) along the RSV genome. The horizontal axis represents four RSV genomes which are banded together. Numbers on the y axis correspond to the frequencies of vsiRNAs mapped to the RSV genomic (−) or antigenomic (+) sequences. D, Schematic genome organization of RSV.

To identify the origin of vsiRNAs in RSV genomes, we aligned vsiRNAs with the RSV genomic and antigenomic sequences. Results of the alignment show that most vsiRNAs identified in *O. sativa* are derived from the RSV genomic-strand RNAs. For *N. benthamiana* and *L. striatellus*, however, the ratio between the genomic-strand derived and the antigenomic-strand derived vsiRNAs is relatively similar (Figure 6). The number of vsiRNAs from the same loci in the genomic- or antigenomic-strand is similarly distributed in *N. benthamiana* and *L. striatellus*, but much more vsiRNAs in *O. sativa* were from the genomic-strand (Figure 7). Although RSV RNA molecules of both polarities are encapsidated in infected cell, they are not encapsidated in an equal molar amounts [37]. Indeed for tenuiviruses, the genomic-strand viral RNAs are considered more abundant than their complementary strand RNAs [18]. It is possible that the vsiRNAs identified in *N. benthamiana* and *L. striatellus* were derived from RSV dsRNAs, while a different origin may exist for vsiRNAs found in rice. It is noteworthy that Yan et al. reported previously that RSV vsiRNAs could derive nearly equally from the viral and complementary strands of RSV RNAs [21]. In that report approximately 10,180 vsiRNAs were identified from the RSV genomic- and antigenomic-strand RNAs. In this study, a much larger number (198,936) of RSV vsiRNAs were accounted. This larger RSV vsiRNA library may provide us with more accurate information on source of the vsiRNA.

**Figure 8:**
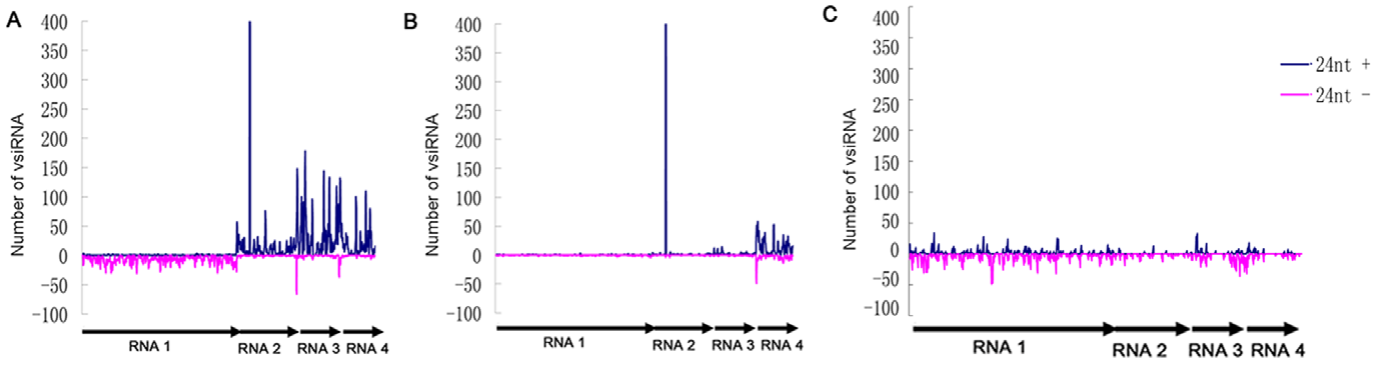
Profile of RSV-derived 24-nt vsiRNAs along the RSV genome from *O. sativa* (A), *N. benthamiana* (B) and *L. striatellus* (C). The 24-nt vsiRNAs matched the genomic and antigenomic sequences are shown in blue and pink, respectively.

Computational analysis of vsiRNA sequences using Perl scripts (Window size = 50-nt and step size = 10-nt) showed that majority RSV vsiRNAs were from the intergenic regions within the RSV genome. Most of the vsiRNAs identified in *O. sativa* were generated from RNAs 3 and 4. In *N. benthamiana*, however, RNA 4 is the major source of vsiRNAs (Figure 8A and 8B). In *L. striatellus*, the vsiRNAs appeared to derive from all four RSV RNA segments, including both coding and intergenic regions (Figure 8C). This finding suggests that recognition of dsRNA substrates by Dicers in plant and insect may be different. One possible scenario is that dicers from plant and *L. striatellus* have different preferences for dsRNA replicative intermediates or highly structured single-stranded RNA molecules. DCLs in plant are somewhat more conserved than those found in insect. Our result presented here indicate that the positions of RSV vsiRNA hot spots are similar in the two plant host tested, indicating that the hot spots may be decided by the virus itself. Alternatively, in plant, the vsiRNAs identified in our libraries may originate from secondary vsiRNAs through amplification by the host RDR pathway. Wang et al demonstrated that hot spots for Cucumber mosaic virus (CMV) vsiRNAs were different in the wild-type and mutant (rdr1 or rdr1-rdr2-rdr6) plants, and these RDRs were known to be involved in amplification of vsiRNAs from CMV genomic RNAs [38]. Thus, RDRs or other factor(s) responsible for generating secondary siRNAs may preferentially amplify vsiRNAs from distinct hot spots within the viral genome in plants. Thirdly, results shown in Figure 3 indicate that the ratios of RSV RNAs 1, 2, 3, and 4 in these two host plants are different from those in *L. striatellus*. For example the ratios between RNAs 4 and 2 in *N. benthamiana* are significantly higher than those in *L. striatellus* (Figure S1). This difference agrees with the result that in *N. benthamiana* the number of hot spots in RNA 4 is enriched compared with that found in RNA4 from *L. striatellus*.

Former works have proved that highly structured regions present in viral RNA genome are also substrates for dicer enzymes in different species [39]. To explore the possibility that the secondary structures of RSV RNAs contribute to the production of vsiRNAs, we evaluated the secondary structures of RSV RNAs 3 and 4, from which most *O. sativa* vsiRNAs were originated, the RNAs 3 and 4 were folded utilizing the RNAfold software as described [40]. Results of the experiment indicate that the potential origin sites of *O. sativa* vsiRNAs are associated with the putative secondary structures in the RSV genomic-strand RNA (Figure S2). Thus, the bias for accumulation of genomic-strand derived vsiRNA in *O. sativa* supports the hypothesis that, like the full length dsRNA replicative intermediates, the highly structured regions within the viral RNA molecules can also serve as substrates for dicer enzymes. The difference between origins of *O. sativa* and *N. benthamiana* vsiRNAs is quite unexpected. The evolutionary adaptation of RSV in *O. sativa* may be responsible for the distinct behaviors of vsiRNAs and the RNAi-mediated antiviral activity in this host.

### Presence of 24-nt vsiRNAs in *O. sativa* and *N. benthamiana*, but not in *L. striatellus*

Further analysis of the 21, 22, and 24-nt vsiRNAs showed that within the RSV genome the distribution peaks representing the 21-nt vsiRNAs were overlapped with the peaks representing the other sized vsiRNAs (Figure 7). For 24-nt vsiRNAs, distribution peaks were observed only for *O. sativa* and *N. benthamiana*. (Figure 8). The highest 24-nt vsiRNA peaks found in the *O. sativa* and *N. benthamiana* libraries are located in the RNA 2 (nucleotide position 778 to 801; TCGAAATGGTGCTACGCACCACAT). The 24-nt vsiRNAs identified at this position accounted for 388 (*O. sativa*) and 497 (*N. benthamiana*) times, respectively. These 24-nt visRNAs also represent approximately 5 (*O. sativa*) and 28% (*N. benthamiana*) of all the 24-nt vsiRNAs identified in these two hosts (Table 1). Nucleotide position 778 to 801 in the RNA2 is located within the intergenic region between the ORF NS2 and NSvc2. A recent report indicated that no potential stem-loop structures were present in this intergenic region [41]. Our result suggests the presence of a unique 24-nt vsiRNA generation mechanism in plant and this mechanism is not present in *L. striatellus*. Further investigations are needed to elucidate the mechanism by which the 24-nt vsiRNAs are made and the role of these 24-nt vsiRNAs in antiviral immunity.

### Dicer 2 and agronaute 2 orthologs in *L. striatellus*

**Figure 9:**
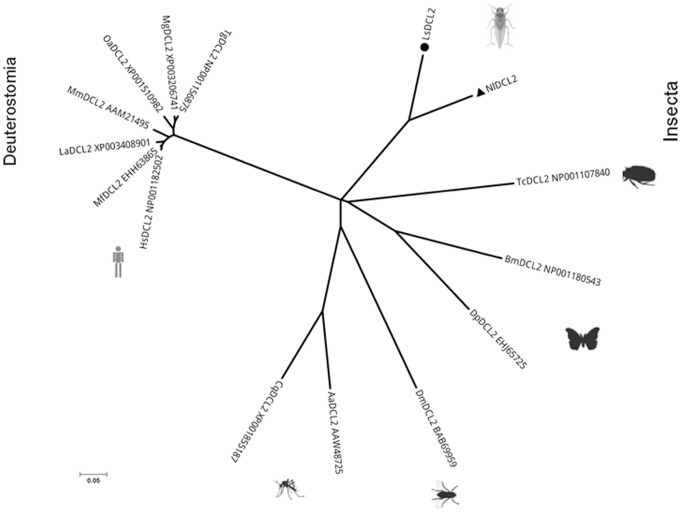
Phylogenetic relationships of Dicer 2 proteins from fifteen insect species. Accession numbers and abbreviations of Dicer 2 proteins in GenBank are as follows: *Culex quinquefasciatus* (CqDCR2, EDS34500.1), *Aedes aegypti* (AaDCR2, AAW48725), *Drosophila melanogaster* (DmDCR2, BAB69959), *Danaus plexippus* (DpDCR2, EHJ65725), *Bombyx mori* (BmDCR2, NP001180543), *Tribolium castaneum* (TcDCR2, NP001107840), *Nilaparvata lugens* (NlDCR2, JX023532), *Laodelphax striatellus* (LsDCR2, JX023531), *Taeniopygia guttata* (TgDCR2, NP001156875), *Meleagris gallopavo* (MgDCR2, XP003206741), *Ornithorhynchus anatinus* (OaDCR2, XP001510982), *Mus musculus* (MmDCR2, AAM21495), *Loxodonta africana* (LaDCR2, XP003408901), *Macaca fascicularis* (MfDCR2, EHH63865), *Homo sapiens* (HsDCR2, NP001182502).

**Figure 10:**
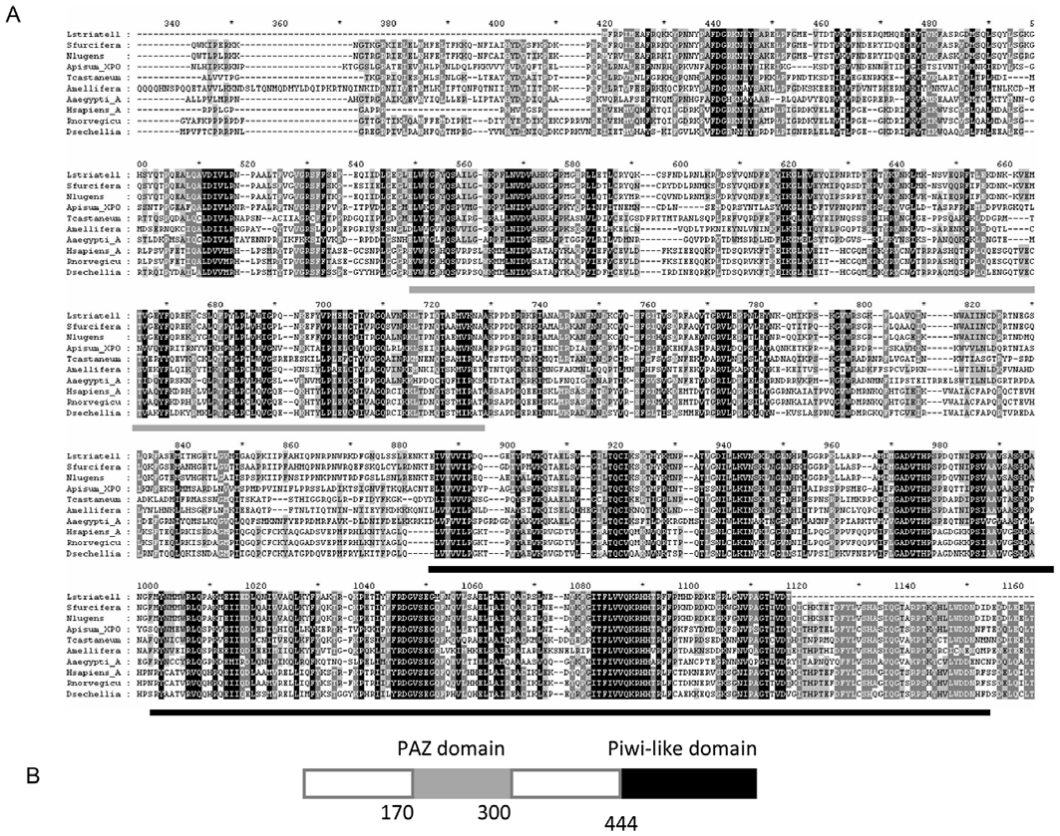
Sequence alignment of Argonaute 2 from *L. striatellus* and other insect species (A), and structure of the partial Argonaute proteins predicted using the NCBI Conserved Domains Server (B). Conserved PAZ and Piwi domains were identified in the assembled *L. striatellus* AGO2. Abbreviation and accession number of Argonaute 2 proteins of different species are as follows: *Laodelphax striatellus* (Lstratell, JX023533), *Sogatella furcifera* (Sfurcifera, JX023535), *Nilaparvata lugens* (Nlugens, JX023534), *Acyrthosiphon pisum* (Apisum_XPO, XP_003240621), *Tribolium castaneum* (Tcastaneum, NP_001107828), *Aedes aegypti* (Aaegypti_A, ACR56327), *Homo sapiens* (Hsapiens_A, NP_001158095), *Rattus norvegicus* (Rnorvegicu, NP_067608), *Drosophila sechellia* (Dsechellia, XP_002045323).

Dcr2 and Ago2 genes are known to be the key elements involved in recognition of dsRNA and targeting viral RNAs for degradation [6]. To determine the function(s) of these genes in *L. striatellus*, we cloned partial Dcr2 and Ago2 orthologs from *L. striatellus*. Phylogenetic analyses using the downloaded Dcr2 sequences and the cloned *L. striatellus* Ago2 ortholog showed that the *L. striatellus* Dcr2 belongs to the same Dcr2 clade for insects (Figure 9), suggesting that the *L. striatellus* Dcr2 may function like the Dcr2 identified from *Drosophila*. The cloned *L. striatellus Ago*2 gene was sequenced and used to blast search the NCBI database. Figure 10 shows that the typical PAZ and Piwi domains reported for other Argonaute family proteins are also present in the *L. striatellus* Ago2. In this study we also identified Ago2 sequences for other two important *O. sativa* planthoppers (*Nilaparvata lugens* and *Sogatella furcifera*), through analysis of published transcriptome data [42], [43]. Sequences of these two Ago2 genes were then used to compare with their orhtologs from other species, including *Homo sapiens, Tribolium castaneum, Acyrthosiphon pisum*, *Aedes aegypti, Apis mellifera, Rattus norvegicus*, and *D. sechellia*. As expected, all the sequences analyzed in this study are conserved (Figure 10A). Among insects, the amino acid sequence identity for the PAZ and Piwi motifs are approximately 40 and 60%, respectively (Figure 10B). For the three closely related planthoppers, the Ago2 amino acid sequence identity is over 84%. This high sequence identity is reasonable because these planthopper species belong to the same family *Delphacidae*, and have similar ecological niches in the rice eco-system.

### Accumulation of RSV in Ago2 repressed *L. striatellus*

**Figure 11:**
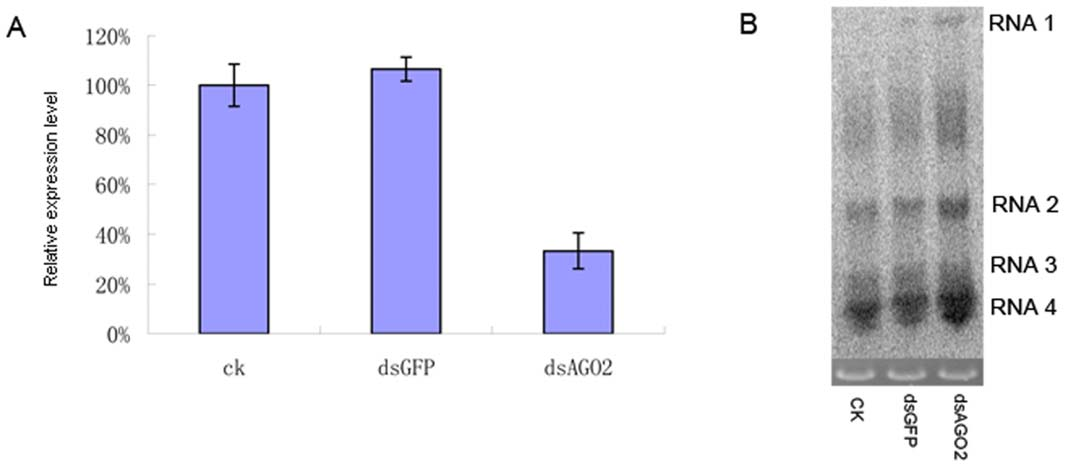
RNAi controlled RSV infection in *L. striatellus*. A: Relative expression level of Ago2 in *L. striatellus* at 6 days post injection with dsRNAs. The experiments were repeated three times. B: Detection of RSV genomic RNA accumulation after silencing *Ago*2 gene. Lane 1, non-treated viruferious *L. striatellus*; lane 2, *L. striatellus* injected with dsGFP; lane 3, *L. striatellus* injected with dsAgo2.

To investigate the role(s) of Ago2 in RNA silencing in *L. striatellus*, 600-bp sense and antisense fragments were prepared individually from the cloned *L. striatellus* Ago2 construct through *in vitro* transcription, annealed together to produce double-stranded RNAs at a concentration of 7 µg/µl, and the dsRNAs were then injected into RSV-viruliferous *L. striatellus*. As shown in Figure 11A, the Ago2 mRNA accumulation in the injected *L. striatellus* was decreased about 70% compared with that in the GFP dsRNA-injected *L. striatellus*. As expected the accumulation of RSV genomic RNAs were significantly increased in the Ago2 dsRNAs-injected *L. striatellus*, as indicated by the Northern blot assay (Figure 11B). This result demonstrates that the Ago2 does play an important role in protecting *L. striatellus* from RSV infection.

In conclusion, we observed abundant accumulation of RSV vsiRNAs in RSV-infected *L. striatellus* and silencing Ago2 in *L. striatellus* enhanced RSV virus accumulation. This finding provides direct and first evidence that RNAi-mediated immunity provides protection against Rice stripe virus in *L. striatellus*. Furthermore, we generated two independent deep sequencing database for RSV-derived vsiRNAs from *O. sativa*, a naturally host of RSV, and *N. benthamiana*, an experimental host of RSV. Our comparative analysis of vsiRNAs from three different libraries revealed that RNA silencing mediated antiviral activity was differently displayed in different RSV hosts.

## Materials and Methods

### Ethics Statement

No specific permits were required for the described field studies.

### Source of virus, host plant and insect vector

RSV used in this study was originally collected from a rice field in Zhejiang Province, China, and maintained in *O. sativa* plants through transmission using *L. striatellus*. Four DNA segments representing RSV RNAs 1, 2, 3, and 4 were individually RT-PCR amplified from purified RSV RNAs using the Phusion High-Fidelity DNA polymerase (New England Biolabs, Ipswich, USA) and cloned followed by sequencing. Sequences of the four fragments are deposited in the GenBank (accession no. JQ927433, JQ927427, JQ927421, JQ927415). These four fragments were used as references for our computational analysis. Primers used to amplify the four genomic segments are listed in the supplemental Table 1. All *O. sativa* plants used in this study were grown inside a growth chamber set at 26±1°C, 80% relative humidity, and a photoperiod of 16 h in light and 8 h in the darkness. *L. striatellus* (small brown planthopper) was originally provided by Institute of Plant Protection, Jiangsu Academy of Agricultural Sciences, China, and was maintained on *O. sativa* cv. Wuyujing No. 3 plants in a culture room set at 25±1°C, 80% relative humidity, and 16/8 hours light/darkness.

### Virus inoculation

*N. benthamiana* plants at 6-to-8 leaf stage were mechanically inoculated with crude extracts from RSV-infected *O. sativa* leaves as described previously [44]. To obtain viruliferous *L. striatellus*, RSV-infected Wuyujing No. 3 seedlings were placed inside a glass beaker followed by adding approximately 200 second instar *L. striatellus* nymphs into the beaker. The beaker was then covered with a nylon mesh to prevent the nymphs from escaping. The juvenile *L. striatellus* were gently transferred onto healthy *O. sativa* seedlings or *N. benthamiana* plants after two day feeding on the RSV-infected Wuyujing No. 3 seedlings. The inoculated *O. sativa* and *N. benthamiana* plants were grown inside a growth chamber under the same conditions as discussed above for 10 days.

### Total RNA extraction and enrichment of low molecular weight (LMW) RNA

Total RNA was extracted from infected *O. sativa*, *N. benthamiana* or *L. striatellus* using TRizol Reagent as instructed (Invitrogen, Carlsbad, USA). Low molecular weight (LMV) RNAs in the total RNA samples were enriched using PEG (molecular weight 8000) and NaCl as described [8]. After adding one volume of loading dye to the LMW RNA samples, they were heated at 95°C for 3 min followed by electrophoresis in a 15% polyacrylamide gel (PAGE) containing 8 M urea and in 0.5× Tris-borate-EDTA buffer. Synthetic DNA oligonucleotides of 18 and 28-nt in length were used as the size markers, and the LMW RNA products were visualized under a UV light source after the SYBR-Gold (Invitrogen) staining. LMW RNAs (sRNAs) of 18∼28-nt in length were excised from gels and used for small RNA (sRNA) library constructions.

### sRNA library construction and Illumina sequencing

sRNA libraries were constructed as described [8]. Briefly, 18∼28-nt sRNAs were sequentially ligated to a 3′ and a 5′ adapter. After each ligation step, sRNAs were purified by electrophoresis using a 15% denaturing PAGE as described above. The final purified ligation products were reverse transcribed into cDNAs using the Superscript III reverse transcriptase (Invitrogen). The first strand cDNAs were PCR amplified using the Taq polymerase (Roche Applied Science, Basel, Switzerland) and specific primers listed in Table S1. DNA amplicons from different libraries were purified individually and submitted to the high-throughput sequencing using the Solexa platform (Illumina, SanDiego, CA).

### Analyses of sRNA sequences

After removal of the adapter sequences and low quality reads, the remaining 18∼28-nt sRNAs were aligned with the four RSV reference sequences described above using the Short Oligonucleotide Analysis Package (SOAP) using the standard parameters for genome assembly [45]. Reads showed zero mismatch with the RSV reference sequences were retained and analyzed using the Perl scripts and Excel as described [22]. The resulting data are deposited at the Status of the NCBI Sequence Read Archive (SRA) (http://www.ncbi.nlm.nih.gov/sra). To predict RSV sequence structure, the thermodynamic prediction of minimal free energy (MFE) method at the RNAfold websites was used [40].

### Northern blot

To detect viral genomic RNAs, 15 µg of total RNA extracted from the RSV-infected rice, *N. benthamiana* and SBPH were separated on 1% (w/v) formaldehyde-denaturing agarose gels and transferred onto Hybond-N+ membranes (Amersham, Buckinghamshire, UK) using a semidry transfer apparatus (Bio-Rad, Hercules, CA) followed by cross-linking using the UV radiation as described (UVP, Upland, CA). The α-32P-dUTP labeled RNA probes were *in vitro* transcribed from the PMD-18T-Rep, PMD-18T-NS2, PMD-18T-NS3 or PMD-18T-SP constructed previously [46] using the Riboprobe System-T7 Kit (Promega, Madison, USA). Primers used in this experiment are listed in Table S1.

### Identification and cloning of Ago2 ortholog from *L. striatellus*

Sequence of *L. striatellus* Argonaute-2 (Ago2) was obtained through searching *L. striatellus* transcriptome database published previously [47] using the *D. melanogaster* Ago2 sequence (GenBank accession No DQ228766.1). A partial Ago2 sequence (containing 1990 nucleotides) was amplified from Oligo (dT) reverse transcripted cDNAs and cloned into the pGEM®-T Easy Vector (Promega). A full length Dicer 2 (Dcr2) sequence was amplified based on the information from the *L. striatellus* transcriptome database followed by 5′Race using 5′ RACE system for rapid amplification of cDNA Ends (Invitrogen) and common PCR. Sequences of Ago2 for other two rice planthoppers (*Nilaparvata lugens* and *Sogatella furcifera*) and of Dcr2 for *N. lugens* were also obtained by searching their transcriptome databases, respectively [42], [43]. Sequences of these Ago2 and Dcr-2 are also deposited in the Genbank and their accession numbers are shown in the annotation presented in Figure 9 and Figure 10. Primers used this experiment are listed in Table S1.

### Phylogenetic and amino acid analysis

Deduced amino acid sequences for different Dicers and Agos in different insect species were aligned using the Clustal W (http://www.clustal.org/clustal2). A phylogenetic tree was constructed using the Neighbour-joining method (NJ) with 1000 bootstrap, resampling the statistics implemented in MEGA 5.0. The aligned sequences were edited and converted into diagrams using software GenDoc(http://gendiapo.sourceforge.net/).

### Construction of RNAi vectors and dsRNA-mediated gene silencing

*L. striatellus* Ago2 dsRNA was synthesized *in vitro* using the Riboprobe System-T7 Kit (Promega). The dsRNA of Green fluorescent protein (GFP) was also made and used as a control. The *in vitro* synthesized Ago2 dsRNA was stained with Bromophenol blue (Sigma-Aldrich, St. Louis, USA) and injected into the thorax of ice-anesthetized third instar *L. striatellus* nymphs using the TransferMan NK 2 micromanipulator as instructed (Eppendorf, Hamburg, Germany). 10 µl dsRNA (7 µg/ul) was used to inject about 250 *L. striatellus* nymphs. Total RNA was extracted from the injected insects at 6 days post injection. Quantitative PCR was carried out on the LightCycler 480@ II using the LightCycler 480@ SYBR I Master kit (Roche Applied Science, Basel, Switzerland). PCR conditions were 95°C for 5 min; followed by 48 cycles at 95°C for 10 s, 60°C for 15 s, and 72°C for 20 s. *L. striatellus* endogenous 18S rRNA gene was used as an internal control for normalization. Primers used in the qRT-PCR for validation of differentially expressed genes are also shown in Table S1.

## Supporting Information

Figure S1 **The ratios between RNAs 4 and 2 in** ***N. benthamiana*** **and** ***L. striatellus*** **.** The value was calculated by Image Quant TL Analysis Tool (GE Company, Fairfield, USA).
(TIF)

Figure S2 **Structural analysis of RSV vsiRNA hot spots in RSV RNAs from** ***O. sativa*** **using RNAfold.** A: The secondary structures of RNAs 3 and 4 were predicted using the thermodynamic prediction of minimal free energy (MFE) (Sui, 2011), a mountain plot representation of the MFE structure is shown. B: Profile of genomic-strand vsiRNAs along the RNAs 3 and 4 sequences. _*_ indicates that vsiRNA production was consistent with the predicted highly structured regions; # indicates that vsiRNA production was discrepant from the predicted secondary structures.
(TIF)

Table S1 **Primers used in Dcr-2 and Ago 2 cloning,** ***in vitro*** **transcription and northern blot.**
(XLS)
