# Supplementary figures and images for: Population Diversity of Rice Stripe Virus-Derived siRNAs in Three Different Hosts and RNAi-Based Antiviral Immunity in Laodelphgax striatellus
Source: PLoS One. 2012 Sep 28;7(9):e46238. doi: 10.1371/journal.pone.0046238 (PMC3460854; doi:10.1371/journal.pone.0046238)

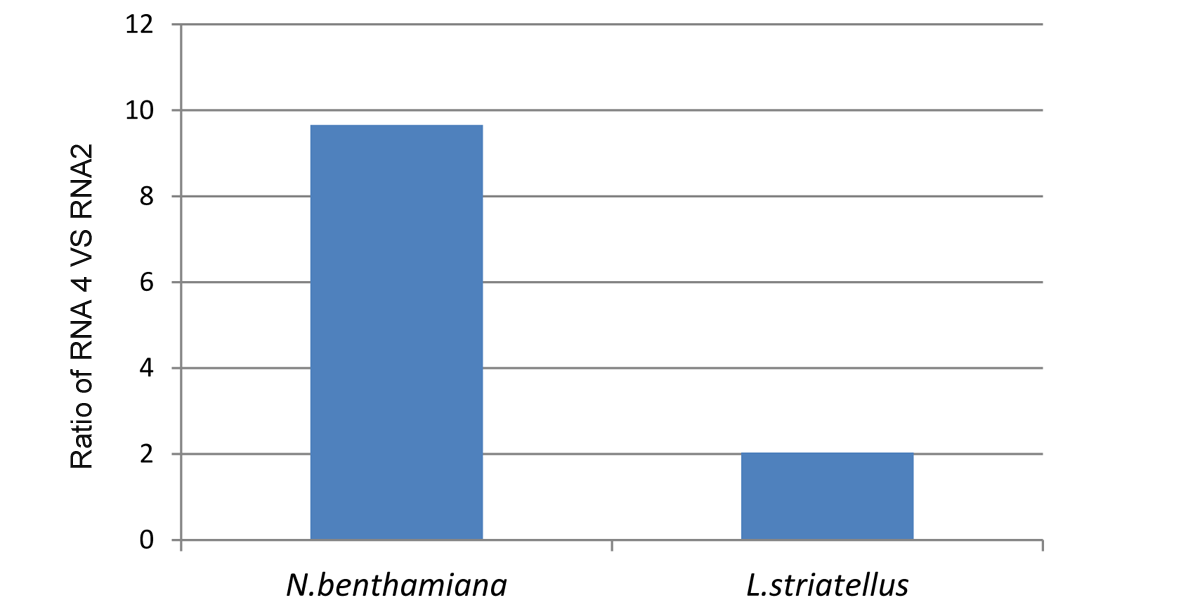

Supplement: Figure S1 — The ratios between RNAs 4 and 2 in N. benthamiana and L. striatellus . The value was calculated by Image Quant TL Analysis Tool (GE Company, Fairfield, USA). (TIF) [file pone.0046238.s001.tif]

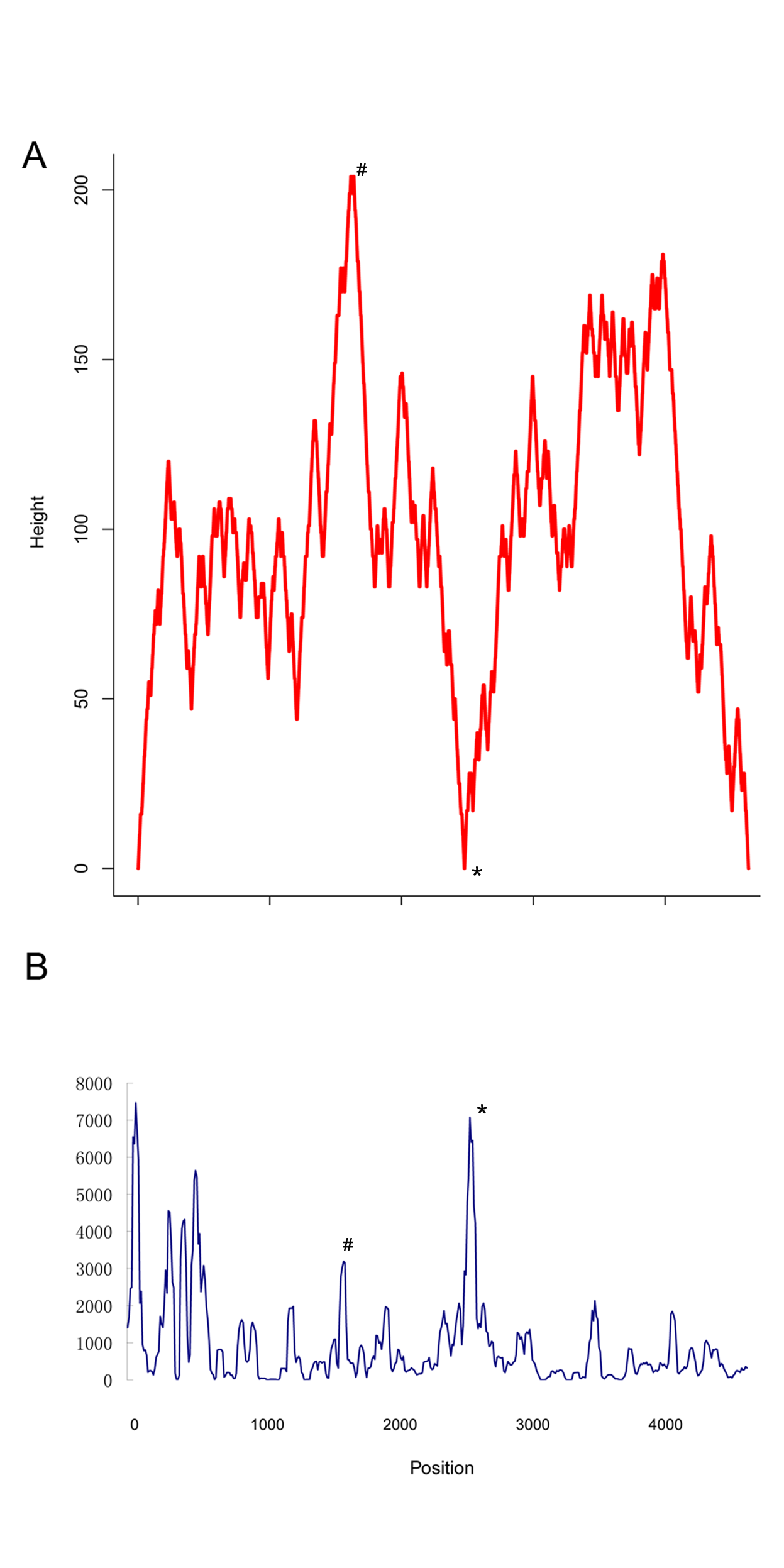

Supplement: Figure S2 — Structural analysis of RSV vsiRNA hot spots in RSV RNAs from O. sativa using RNAfold. A: The secondary structures of RNAs 3 and 4 were predicted using the thermodynamic prediction of minimal free energy (MFE) (Sui, 2011), a mountain plot representation of the MFE structure is shown. B: Profile of genomic-strand vsiRNAs along the RNAs 3 and 4 sequences. * indicates that vsiRNA production was consistent with the predicted highly structured regions; # indicates that vsiRNA production was discrepant from the predicted secondary structures. (TIF) [file pone.0046238.s002.tif]
